# Amorphigenin联合顺铂对人肺腺癌A549/DDP细胞的协同抗肿瘤作用

**DOI:** 10.3779/j.issn.1009-3419.2016.12.02

**Published:** 2016-12-20

**Authors:** 红珍 钟, 瑜芳 左, 鑫 巫, 艳 彭, 会萍 何, 俊 杨, 成浓 官, 祖敏 徐

**Affiliations:** 1 524000 湛江，广东医科大学附属医院肿瘤中心 Cancer Center, Affiliated Hospital of Guangdong Medical University, Zhanjiang 524000, China; 2 524000 湛江，广东医科大学天然药物研究与开发重点实验室 Guangdong Key Laboratory for Research and Development of Natural Drugs, Guangdong Medical University, Zhanjiang 524000, China

**Keywords:** Amorphigenin, 顺铂, 肺腺癌, 凋亡, 抗肿瘤作用, Amorphigenin, Cisplatin, Lung adenocarcinoma, Apoptosis, Antitumor effect

## Abstract

**背景与目的:**

Amorphigenin是从紫穗槐属植物的种子中分离提取的鱼藤酮类化合物，研究发现amorphigenin对多种肿瘤细胞具有增殖抑制作用。本研究拟探讨amorphigenin对人肺腺癌耐顺铂细胞株A549/DDP的抗肿瘤作用及其可能的分子机制。

**方法:**

采用CCK-8法测定A549/DDP细胞的增殖；克隆形成实验测定A549/DDP细胞的克隆形成；流式细胞术检测细胞的凋亡率；Western blot技术检测caspase-3、PARP和LRP蛋白的表达。

**结果:**

Amorphigenin可抑制A549/DDP细胞的增殖48 h[半数抑制浓度(half maximal inhibitory concentration, IC_50_)]为(2.19±0.92)μmol/L、抑制克隆形成及诱导细胞凋亡。此外，Amorphigenin与顺铂联合可协同地抑制A549/DDP细胞生长和促进凋亡；降低耐药蛋白LRP蛋白的表达。

**结论:**

Amorphigenin可抑制A549/DDP细胞增殖和促进细胞凋亡；amorphigenin可能是通过抑制耐药蛋白LRP蛋白表达，进而与顺铂对A549/DDP细胞产生协同抑制作用。

## Abstract (en)

**Background and objective:**

Amorphigenin, a rotenoid compouns, from seeds of Amorpha fruticosa, has been shown to possess anti-proliferation activities in several cancer cells. To explore the antitumor effects of amorphigenin on cisplatin-resistant human lung adenocarcinoma A549/DDP cells and explore the underlying mechanisms.

**Methods:**

CCK-8 assay was used to measure the proliferation of A549/DDP cells; Colony formation assay was used to measure the colony formation of A549/DDP cells; Flow cytometry assay was used to detect the apoptosis rates; Western blot analysis was used to explore the expression of apoptosis-related proteins (caspase-3 protein, PARP protein) and lung resistance protein (LRP).

**Results:**

Our results demonstrated that amorphigenin could inhibit the proliferation of A549/DDP cells with a inhibition concentration of 50% cell growth (IC_50_) at 48 h of (2.19±0.92) μmol/L. Amorphigenin could inhibit the colony formation ability and induce apoptosis of A549/DDP cells; Furthermore, amorphigenin combined with cisplatin showed synergistic proliferation-inhibitory effect and apoptosis-promoting effect in A549/DDP cells; reduced the expression of LRP of A549/DDP cells.

**Conclusions:**

Amorphigenin remarkably inhibits the proliferation and induces apoptosis in A549/DDP cells. Combination of amorphigenin with cisplatin had the synergistic inhibitory effect on A549/DDP cells by downregulating the expression of LRP.

目前肺癌发病率、死亡率居首位^[1]^。临床上通常将肺癌分为小细胞肺癌和非小细胞肺癌，非小细胞肺癌占85%^[2]^。而肺癌的化学治疗主要采用顺铂联合吉西他滨、紫杉醇等当中的一种^[3]^。虽然化疗在肺癌的治疗中取得了一定的进展，但5年总生存率仍较差，低于16%^[4]^。顺铂耐药是影响肺癌化疗疗效的重要因素之一。因此，克服肺癌对顺铂的耐药具有重要的意义。Amorphigenin是从紫穗槐的种子中提取得到的一种鱼藤酮类化合物^[5]^。研究^[6-8]^发现amorphigenin有多种生物活性，如抗增殖作用、护肝作用、抑制神经氨酸酶的作用及抑制肺癌、结肠癌、黑色素瘤、口腔癌，白血病、前列腺癌、乳腺癌等细胞的生长。虽然amorphigenin可抑制人肺腺癌细胞A549^[7]^和人肺癌细胞Lu-1^[8]^的生长，但amorphigenin对耐顺铂肺腺癌A549/DDP细胞的作用及抗肿瘤作用的机制目前在国内外尚未见报道。本研究的目的是研究amorphigenin对人肺腺癌耐顺铂细胞株A549/DDP的抗肿瘤作用并探讨其可能的分子机制。

## 1 材料与方法

### 1.1 主要试剂

Amorphigenin由广东医科大学天然药物研究与开发重点实验室提供，纯度 > 95%^[5]^。顺铂购于江苏豪森药业股份有限公司，二甲亚砜(DMSO)从美国MP Biomedicals LLC公司购买，RPMI-1640培养基、胎牛血清(FBS)购买于美国Gibco公司，胰酶购于吉诺生物医药技术有限公司，碘化丙锭(PI)和FITC Annexin V凋亡检测试剂盒购自于美国BD公司，兔抗人PARP、caspase-3、LRP抗体购买于Sigma公司。辣根酶标记的山羊抗兔IgG、鼠抗人GAPDH多克隆抗体均购买于碧云天公司。Amorphigenin用DMSO溶解，配制成50 μmol/L的储存液，-20 ℃冰箱贮存。实验前用RPMI-1640培养基稀释，将DMSO的终浓度控制在 < 0.1%。

### 1.2 细胞培养

人肺腺癌顺铂耐药细胞株A549/DDP和A549细胞由本实验室保存并常规传代培养，培养于含10%胎牛血清、青霉素(100 U/mL)和链霉素(100 U/mL)的RPMI-1640培养基中，在37 ℃、5%CO2、饱和湿度的培养箱内培养。细胞每2天-3天常规传代一次，所有实验的细胞均使用对数生长期的细胞。

### 1.3 CCK-8法测定细胞活力

取对数生长期的细胞，常规胰酶消化成单个细胞悬液，按5.0×10^3^个细胞/孔的密度种于96孔板中，置于培养箱中孵育过夜后，分别加入终浓度为0.5 μmol/L、1 μmol/L、2 μmol/L、4 μmol/L、8 μmol/L、16 μmol/L的amorphigenin或终浓度为2.5 μg/mL、5 μg/mL、10 μg/mL、20 μg/mL、40 μg/mL的顺铂。于培养箱培养24 h、48 h后，弃去原来的培养液，然后每孔加入按说明书新配制的100 μL CCK-8试剂，再于培养箱孵育2 h后，用酶标仪于450 nm波长处测吸光值。按照下列公式计算药物对细胞的存活率：存活率%=实验组吸光值/对照组吸光值×100，并计算48 h的半数抑制浓度(half maximal inhibitory concentration, IC_50_)。

### 1.4 克隆形成实验

取对数生长期的细胞，消化成单个细胞悬浮液，500个细胞/孔接种于6 cm培养皿中，待细胞生长稳定时，加入终浓度为0.062, 5 μmol/L、0.125 μmol/L、0.25 μmol/L的amorphigenin，空白对照组加入等量的培养基。放置培养箱培养15 d，当形成肉眼可见的细胞克隆后终止培养，用PBS洗2次，再用甲醇固定，然后用结晶紫染色，洗净晾干，在显微镜下拍照计数。克隆形成率%=(实验组克隆数/对照组克隆数)×100%。

### 1.5 联合指数计算

我们使用Chou-Talalay方法研究药物组合的协同的可能性^[9]^。取对数生长期的细胞，常规胰酶消化成单个细胞悬液，按5.0×10^3^个细胞/孔的密度种于96孔板中，置于培养箱中孵育过夜后，除去原来的培养基；先用浓度为0.5 μmol/L、1 μmol/L的amorphigenin处理人肺腺癌耐顺铂细胞株A549/DDP 24 h后；再用浓度为2.5 μg/mL、5 μg/mL、10 μg/mL、20 μg/mL、40 μg/mL的顺铂处理24 h；弃去原来的培养液，然后每孔加入按说明书新配制的100 μL CCK8试剂，再于培养箱孵育2 h后，用酶标仪于450 nm波长处测吸光值。按照下列公式计算细胞的存活率：存活率%=实验组吸光值/对照组吸光值×100。使用CompuSyn软件自动计算出两药的联合指数及描绘出两药等效图，在等效图中(D_1_/D_x_1__)为横坐标，(D_2_/D_x_2__)为纵坐标，D_1_、D_2_为两药合用产生x效应时两药各种所需的浓度，而D_x_1__、D_x_2__则为两药单独使用时产生x效应时两药各自的浓度；根据Chou-Talalay定理规定CI < 1则两药联合为协同作用，CI=1则为相加作用，CI > 1，则为拮抗^[10]^。

### 1.6 细胞凋亡测定

取对数生长期的细胞，消化成单个细胞悬液，8×10^3^个细胞/孔，接种于6孔板，置于培养箱孵育过夜，加入终浓度为0.5 μmol/L、1 μmol/L、2 μmol/L、4 μmol/L、8 μmol/L、16 μmol/L的amorphigenin，联合组amorphigenin浓度为0.5 μmol/L和顺铂浓度为10 μg/mL，处理48 h后，消化收集细胞，用PI和FITC Annexin V染色，以流式细胞术检测细胞凋亡率，未处理组细胞为对照组。

### 1.7 免疫印迹实验

取对数生长期的细胞，以2×10^5^个/孔的密度接种于6 cm培养皿中，孵育过夜后按以下方法加入药物：amorphigenin组(0.5 μmol/L)、顺铂组(10 μg/mL)、amorphigenin联合顺铂组，处理48 h后，提取细胞总蛋白，经BCA法测定蛋白浓度，取12 μg总蛋白上样，十二烷基硫酸钠-聚丙烯酰胺凝胶电泳(SDS-PAGE)，再转移到PVDF膜上，5%脱脂奶粉室温封闭2 h，加入一抗于4 ℃冰箱孵育过夜，辣根过氧化酶标记的二抗室温孵育1h，洗膜后辣根过氧化物酶HRP-ECL发光显色法对膜进行显色曝光。

### 1.8 统计学方法

数据采用GraphPad Prism 5软件进行单因素方差分析或*t*检验，所有数据均为3次独立实验结果，以Mean±SD表示。*P* < 0.05为差异有统计学意义。

## 2 结果

### 2.1 Amorphigenin对人肺腺癌细胞株A549及耐顺铂株A549/DDP的生长抑制作用

CCK-8结果显示顺铂能抑制A549/DDP和A549细胞的生长，A549/DDP的48 h的IC_50_为(16.91±1.60)μmol/L，而A549的48 h的IC_50_为(2.84±0.18)μmol/L，A549/DDP的耐药倍数约为9.7倍(图 1A)，表明A549/DDP对顺铂具有明显的耐药性。此外，amorphigenin呈浓度依赖性地抑制耐顺铂株A549/DDP的生长(图 1B)，其48 h的IC_50_为(2.19±0.92)μmol/L。这些结果表明amorphigenin对人肺腺癌耐顺铂细胞株A549/DDP有明显的增殖抑制作用。

**1:**
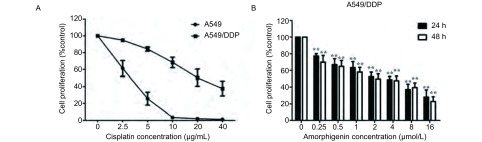
Amorphigenin对肺腺癌耐顺铂细胞A549/DDP的增殖抑制作用。A：不同浓度的顺铂作用于A549细胞和A549/DDP细胞48 h；B：Amorphigenin作用于A549/DDP细胞24 h或者48 h。^**^：*P* < 0.01，与对照组比较。 Amorphigenin could significantly inhibit the proliferation in cisplatin-resistant human lung adenocarcinoma A549/DDP cells. A: A549 and A549/DDP cells were treated with increasing concentrations of cisplatin for 48 h; B: A549/DDP was treated with increasing concentrations of amorphigenin for 24 h or 48 h. ^**^: *P* < 0.01, compared with the control group.

### 2.2 Amorphigenin抑制人肺腺癌耐顺铂细胞株A549/DDP的克隆形成

分别用0.062, 5 μmol/L、0.125 μmol/L、0.25 μmol/L的amorphigenin处理人肺腺癌耐顺铂细胞株A549/DDP 15 d，形成的克隆数目随浓度增加而逐渐减少(图 2A)。统计学分析发现0.062, 5 μmol/L、0.125 μmol/L、0.25 μmol/L的amorphigenin作用于A549/DDP细胞时，克隆形成率分别为(84.31±4.46)%、(42.49±8.65)%和(9.94±5.89)%(图 2B)。这提示amorphigenin能以浓度依赖方式抑制人肺腺癌耐顺铂细胞株A549/DDP的克隆形成。

**2:**
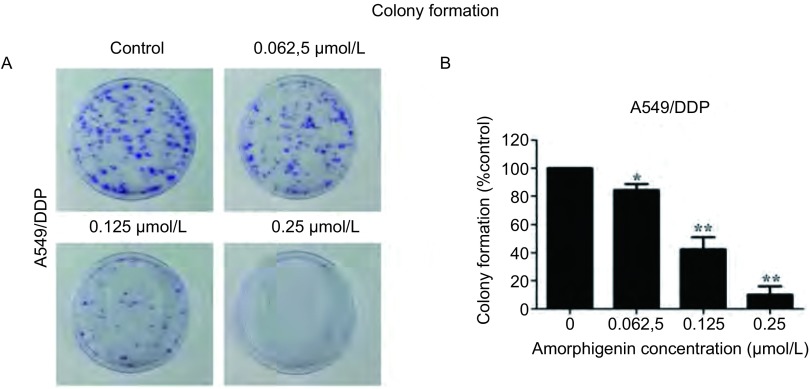
Amorphigenin抑制肺腺癌耐顺铂细胞A549/DDP的克隆形成。A：细胞培养15天后，用结晶紫染色。B：在显微镜下统计细胞克隆数目。^*^：*P* < 0.05，^**^：*P* < 0.01，与对照组比较。 Amorphigenin inhibits the colony formation of cisplatin-resistant human lung adenocarcinoma A549/DDP cells. A: Colonies were stained with crystal violet at 15 days after culture; B: The number of colonies was counted under microscope. ^*^: *P* < 0.05, ^**^: *P* < 0.01, compared with the control group.

### 2.3 Amorphigenin诱导人肺腺癌耐顺铂细胞株A549/DDP凋亡

我们采用了流式细胞术检测了amorphigenin处理后A549/DDP细胞的凋亡率。用0.5 μmol/L-16 μmol/L浓度的amorphigenin处理人肺腺癌耐顺铂细胞株A549/DDP 48 h，采用PI和FITC Annexin V双染法及流式细胞术检测了细胞的凋亡率，结果显示amorphigenin呈浓度依赖性地诱导A549/DDP细胞的凋亡，当amorphihenin浓度为0.5 μmol/L、1 μmol/L、2 μmol/L、4 μmol/L、8 μmol/L、16 μmol/L时，凋亡率分别为(7.50±1.70)%、(9.20±0.56)%、(13.13±2.24)%、(13.7±4.62)%、(28.93±8.17)%和(69.53±10.52)%(图 3A，图 3B)。为了更进一步说明凋亡途径的激活可能与amorphigenin诱导的细胞凋亡有关，我们采用免疫印迹技术检测了凋亡相关蛋白PARP的表达。结果发现，随着amorphigenin浓度的升高，PARP的蛋白表达水平逐渐减少，而cleaved PARP的蛋白表达水平逐渐增加，提示amorphigenin可能是通过激活PARP通路从而诱导人肺腺癌耐顺铂细胞株A549/DDP细胞凋亡(图 3C，图 3D)。这些结果证明了amorphigenin能诱导人肺腺癌耐顺铂细胞株A549/DDP凋亡。

**3:**
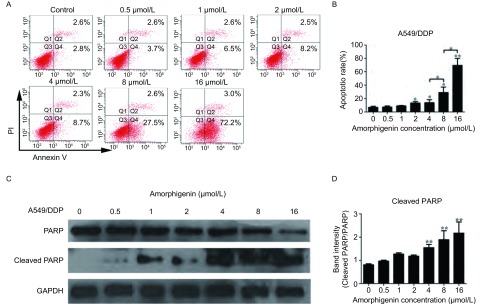
Amorphigenin诱导肺腺癌细胞株A549/DDP凋亡。A、B：Amorphigenin处理A549/DDP细胞48 h；C、D：Amorphigenin对PARP和cleaved PARP蛋白的影响。^*^：*P* < 0.05，^**^：*P* < 0.01，与对照组比较。 Amorphigenin could induce apoptosis in A549/DDP cells. A and B: A549/DDP cells were treated with increasing concentrations of amorphigenin for 48 h; C and D: The levels of PARP and cleaved PARP. ^*^: *P* < 0.05, ^**^: *P* < 0.01, compared with the control group.

### 2.4 Amorphigenin联合顺铂对人肺腺癌耐顺铂细胞株A549/DDP具有协同的抗肿瘤作用

我们先用浓度为0.5 μmol/L、1 μmol/L的amorphigenin处理人肺腺癌耐顺铂细胞株A549/DDP 24 h后，再用浓度为2.5 μg/mL、5 μg/mL、10 μg/mL、20 μg/mL、40 μg/mL的顺铂处理24 h，然后采用CCK-8法检测细胞的增殖变化。结果发现顺铂与0.5 μmol/L、1 μmol/L的amorphigenin联合处理后，顺铂的24 h的IC_50_分别为(15.86±2.91)μg/mL、(5.18±1.94)μg/mL，较单独应用顺铂时(37.99±10.63)μg/mL明显减低(图 4)。这表明amorphigenin能增强顺铂对人肺腺癌耐顺铂细胞株A549/DDP的生长抑制作用。且根据等效线图分析示: Amorphigenin和顺铂10个组合浓度中，9个组合的浓度的CI < 1，1个组合浓度CI > 1；具体CI值：当浓度为0.50 μmol/L的amorphigenin联合2.5 μg/mL的顺铂时CI为1.31，提示拮抗作用；0.50 μmol/L的amorphigenin联合5 μg/mL、10 μg/mL、20 μg/mL、40 μg/mL的顺铂时CI分别为0.97、0.77、0.75、0.82，提示是协同作用；同样浓度为1.00 μmol/L的amorphigenin联合5 μg/mL、10 μg/mL、20 μg/mL、40 μg/mL的顺铂时CI分别为0.60、0.52、0.58、0.60、0.66，提示是协同作用(图 5A，图 5B)。这提示两药联合呈协同作用。

**4:**
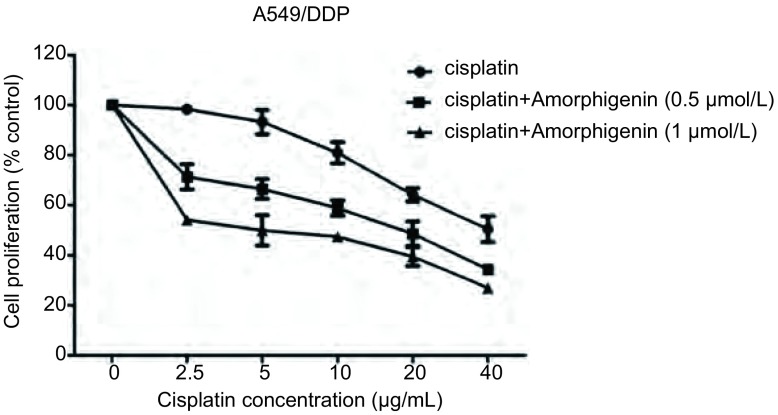
Amorphigenin联合顺铂增强对人肺腺癌耐顺铂细胞株A549/DDP的增殖抑制作用 Combination of amorphigenin with cisplatin enhances the inhibition effects on cisplatin-resistant lung cancer cells

**5:**
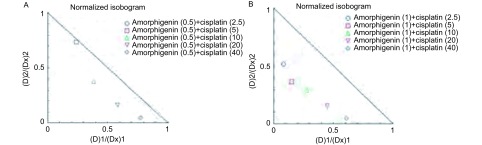
Amorphigenin联合顺铂对人肺腺癌A549/DDP细胞的协同抗肿瘤作用。浓度为0.5 *μ*mol/L(A)和1 *μ*mol/L(B)的amorphigenin联合浓度为2.5 *μ*g/mL、5 *μ*g/mL、10 *μ*g/mL、20 *μ*g/mL、40 *μ*g/mL的顺铂的CI值。根据Chou-Talalay定理规定CI < 1则两药联合为协同作用，CI=1则为相加作用，CI > 1，则为拮抗。 Synergistic antitumor effect of amorphigenin combined wih cisplatin in human lung adenocarcinoma cell A549/DDP. CI values for amorphigenin at 0.5 *μ*mol/L (A) and 1 *μ*mol/L (B) in combination with 2.5 *μ*g/mL, 5 *μ*g/mL, 10 *μ*g/mL, 20 *μ*g/mL, 40 *μ*g/mL cisplatin against A549/DDP cells. CI is a quantitative definition for synergism where CI < 1, additive effect where CI=1 and antagonism where CI > 1.

### 2.5 Amorphigenin增强顺铂对人肺腺癌耐顺铂细胞株A549/DDP的凋亡作用

我们采用流式细胞术测定了两药联合时对人肺腺癌耐顺铂细胞株A549/DDP凋亡的作用，单用amorphigenin时凋亡率为(10.40±1.77)%，单用顺铂时凋亡率为(43.90±6.22)%，两药联合时凋亡率则达到(75.27±8.29)%(图 6A，图 6B)。此外我们还采用了免疫印迹技术检测caspase-3、PARP蛋白表达。结果发现，amorphigenin联合顺铂与单用amorphigenin或顺铂相比，pro-caspase-3、PARP的蛋白表达明显减少，cleaved PARP和cleaved caspase-3的蛋白表达明显增加(图 6C-图 6F)。总之，amorphigenin增强顺铂对人肺腺癌耐顺铂细胞株A549/DDP的凋亡作用。

**6:**
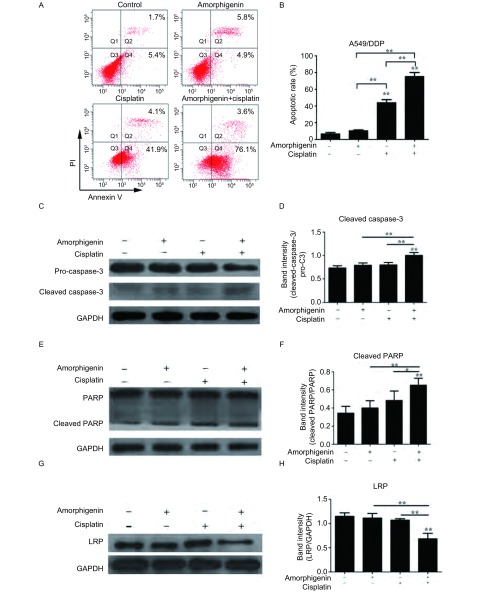
Amorphigenin增强顺铂对人肺腺癌耐顺铂细胞株A549/DDP的凋亡作用。A、B：分别用amorphigenin或/和顺铂处理A549/DDP细胞48 h；C、E和G：Caspase-3、PARP和LRP的Western blot检测结果；D、F和H：Caspase-3、PARP和LRP含量变化的柱状分析图。^*^：*P* < 0.05，^**^：*P* < 0.01，与对照组比较。 Combination of amorphigenin with cisplatin enhances the antitumor effects on A549/DDP cells. A and B: A549/DDP cells were treated with amorphigenin or cisplatin alone or combined with amorphigenin (0.5 *μ*mol/L) and cisplatin (10 *μ*g/mL) for 48 h; C, E and G: The expression of caspase-3, PARP and LRP detected by Western blot; D, F and H: The densitometric analysis of caspase-3、PARP and LRP. ^**^: *P* < 0.01, compared with the control group.

### 2.6 Amorphigenin降低LRP蛋白的表达

分别用amorphigenin，顺铂或amorphigenin联合顺铂处理A549/DDP细胞48 h，采用免疫印迹技术检测LRP蛋白的表达。我们的结果发现，amorphigenin联合顺铂与单用amorphigenin或顺铂相比，LRP蛋白的表达明显减少，而单用顺铂组或amorphigenin组与对照组比较相差不大(图 6G和图 6H)。这提示了amorphigenin通过下调LRP蛋白的表达联合顺铂对肺腺癌耐顺铂细胞株A549/DDP的协同抗肿瘤作用。

## 3 讨论

Amorphigenin是一种鱼藤酮类化合物糖苷类紫穗槐苷的糖基苷元，早期的研究^[11-13]^发现其具有抑制破骨细胞的分化和溶解、保护肝脏、抑制细菌神经氨酸苷酶的作用。进一步的研究发现，amorphigenin可抑制多种肿瘤细胞的增殖作用，如肺腺癌细胞A549、人结肠癌细胞HCT-8、黑色素瘤细胞RPMI-7951、人乙酰胆碱受体细胞TE671、人口腔表皮样癌细胞KB、鼠白血病细胞P388，它们的半数有效量(50% effective dose, ED50)分别为0.05 μg/mL、0.03 μg/mL、0.05 μg/mL、 < 0.01 μg/mL、0.04 μg/mL、0.04 μg/mL^[7]^。人肺癌细胞Lu-1、人激素依赖型前列腺癌细胞LNCaP、人乳腺癌细胞MCF-7，其ED50分别为4.8 μg/mL、7.9 μg/mL和 > 20 μg/mL^[8]^。而我们的研究首次发现amorphigenin以浓度依赖性方式抑制人肺腺癌耐顺铂细胞株A549/DDP细胞的生长，其48 h的IC_50_为(2.19±0.92)μmol/L，并且诱导A549/DDP细胞凋亡。进一步研究的发现amorphigenin诱导人肺腺癌耐顺铂细胞株A549/DDP细胞凋亡的机制可能是通过激活PARP途径。

为了明确amorphigenin能否联合顺铂对人肺腺癌耐顺铂细胞株A549/DDP有协同的抗肿瘤作用。结果发现顺铂与amorphigenin联合处理后，amorphigenin能显著增强顺铂对A549/DDP的生长抑制作用，且amorphigenin能增加顺铂对人肺腺癌耐顺铂细胞株A549/DDP的凋亡率。进一步的研究发现，与amorphigenin或顺铂处理组相比，amorphigenin联合顺铂组pro-caspase-3、PARP蛋白的表达明显降低，而cleaved caspase-3和cleaved PARP蛋白表达明显增加，提示amorphigenin可能通过激活caspase-3、PARP蛋白从而诱导细胞凋亡。

目前的研究认为肺癌顺铂耐药机制主要是以下几个方面：①细胞内药物浓度下降；②细胞解毒功能增强；③DNA损伤修复功能异常；④逃避细胞凋亡^[14]^。细胞内药物浓度下降主要与药物耐药蛋白相关；一些报道的耐药蛋白有肺耐药蛋白(lung resistance protein, LRP)、乳腺癌耐药蛋白(breast cncaer resistance protein, BCRP)、多药耐药相关蛋白(multidrug resistance-associated protein, MRP)；此外，切除修复交叉互补基因1(excision repair cross-completion 1, ERCC1)与顺铂的耐药也密切相关。MRP，是一个ATP依赖的膜运输蛋白，当抗肿瘤药物进入肿瘤细胞时，MRP使用ATP水解的能量把药物泵出细胞，从而减少细胞内的药物浓度增加药物的耐药性。LRP又叫主穹窿蛋白，主要参与细胞内毒性药物的分布而增加耐药，多项研究^[15, 16]^表明肺LRP的高表达会导致对顺铂的耐药。ERCC1、DNA修复核酸内切酶，具有5′DNA核酸内切酶活性，在DNA切除修复过程中能够识别和清除铂类药物诱导的DNA络合物，从而导致对顺铂的耐药。BCRP，属于ABC膜转运蛋白超家族成员，也是依赖ATP将化疗药物泵出细胞，降低细胞内药物浓度，从而增加药物的耐药性^[17]^。Gyemant等^[18]^研究发现amorphigenin通过降低MDR的表达水平来抑制多药耐药的人乳腺癌细胞HTB-26和多药耐药的小鼠淋巴瘤细胞L5178Y的细胞增殖，并且amorphigenin联合表柔比星对多药耐药的小鼠淋巴瘤细胞L5178Y有协同的抗肿瘤作用。而amorphigenin对其他肿瘤的作用机制目前国内外均未见报道。但我们的研究首次发现amorphigenin通过降低LRP蛋白表达的水平来抑制人肺腺癌耐顺铂细胞株A549/DDP的生长，并且amorphigenin联合顺铂对A549/DDP细胞有协同的抗肿瘤作用。本研究我们发现amorphigenin可通过诱导凋亡抑制A549/DDP细胞增殖及增强顺铂对A549/DDP细胞的生长抑制作用。且amorphigenin可通过下调LRP蛋白表达的水平来抑制人肺腺癌耐顺铂细胞株A549/DDP的生长。然而，amorphigenin能否通过影响其他耐药蛋白和/或DNA损伤修复等信号通路，仍需进一步的探索。

总之，本研究发现amorphigenin可通过诱导细胞凋亡抑制人肺腺癌耐顺铂细胞株A549/DDP的生长。Amorphigenin可能是通过抑制耐药蛋白LRP蛋白表达，进而与顺铂对A549/DDP细胞产生协同抗肿瘤作用，这为amorphigenin用于顺铂耐药肺癌的治疗提供了科学依据及理论基础。

## References

[1] Siegel RL, Miller KD, Jemal A (2015). Cancer statistics, 2015. CA Cancer J Clin.

[2] Molina JR, Yang P, Cassivi SD (2008). Non-small cell lung cancer: epidemiology, risk factors, treatment, and survivorship. Mayo Clin Proc.

[3] Wang Y, Chen L, Huang G (2013). Klotho sensitizes human lung cancer cell line to cisplatin via PI3k/Akt pathway. PLoS One.

[4] Jia Y, Zang A, Jiao S (2016). The interleukin-18 gene promoter-607 A/C polymorphism contributes to non-small-cell lung cancer risk in a Chinese population. Onco Targets Ther.

[5] Wu X, Liao H, Wu K (2016). Chemical constituents from the seeds of amorpha fruticosa and their chemotaxonomic significance. OALib.

[6] Ji M, Liang Y, Gu Z (2015). Inhibitory effects of amorphigenin on the mitochondrial complex Ⅰ of culex pipiens pallens coquillett (Diptera: Culicidae). Int J Mol Sci.

[7] Li L, Wang HK, Chang JJ (1993). Antitumor agents, 138. Rotenoids and isoflavones as cytotoxic constitutents from Amorpha fruticosa. J Nat Prod.

[8] Chin YW, Mdee LK, Mbwambo ZH (2006). Prenylated flavonoids from the root bark of Berchemia discolor, a Tanzanian medicinal plant. J Nat Prod.

[9] Chou TC (2010). Drug combination studies and their synergy quantification using the Chou-Talalay method. Cancer Res.

[10] Zhang N, Fu JN, Chou TC (2016). Synergistic combination of microtubule targeting anticancer fludelone with cytoprotective panaxytriol derived from panax ginseng against MX-1 cells *in vitro*: experimental design and data analysis using the combination index method. Am J Cancer Res.

[11] Kim BG, Kwak HB, Choi E (2010). Amorphigenin inhibits Osteoclast differentiation by suppressing c-Fos and nuclear factor of activated T cells. Anatomy Cell Biol.

[12] Kloutek E, Popov A, Drenska D (1985). Experimental research on the hepatoprotective activity of flavonoids isolated from Amorpha fructiosa. Eksp Med Morfol.

[13] Kim YS, Ryu YB, Curtis-Long MJ (2011). Flavanones and rotenoids from the roots of Amorpha fruticosa L. that inhibit bacterial neuraminidase. Food Chem Toxicol.

[14] Florea AM, Busselberg D (2011). Cisplatin as an anti-tumor drug: cellular mechanisms of activity, drug resistance and induced side effects. Cancers (Basel).

[15] Zhang W, Zhou H, Yu Y (2016). Combination of gambogic acid with cisplatin enhances the antitumor effects on cisplatin-resistant lung cancer cells by downregulating MRP2 and LRP expression. Onco Targets Ther.

[16] Li K, Chen B, Xu L (2013). Reversal of multidrug resistance by cisplatin-loaded magnetic Fe3O4 nanoparticles in A549/DDP lung cancer cells *in vitro* and *in vivo*. Int J Nanomed.

[17] Bharthuar A, Saif UR, Black JD (2014). Breast cancer resistance protein (BCRP) and excision repair cross complement-1 (ERCC1) expression in esophageal cancers and response to cisplatin and irinotecan based chemotherapy. J Gastrointest Oncol.

[18] Gyemant N, Tanaka M, Antus S (2005). *In vitro* search for synergy between flavonoids and epirubicin on multidrug-resistant cancer cells. In Vivo.

